# Anti-cancer properties of quercetin in osteosarcoma

**DOI:** 10.1186/s12935-021-02067-8

**Published:** 2021-07-05

**Authors:** Parisa Maleki Dana, Fatemeh Sadoughi, Zatollah Asemi, Bahman Yousefi

**Affiliations:** 1 Research Center for Biochemistry and Nutrition in Metabolic Diseases, Institute for Basic Sciences, Kashan University of Medical Sciences, Kashan, I.R. of Iran; 2 Stem Cell Research Center, Tabriz University of Medical Sciences, Tabriz, Iran; 3 Department of Biochemistry, Faculty of Medicine, Tabriz University of Medical Sciences, Tabriz, Iran

**Keywords:** Quercetin, Osteosarcoma, Apoptosis, Proliferation, Cell viability

## Abstract

Osteosarcoma is a primary bone tumor. Although it is a rare disease in general, it is the most common primary bone tumor among children. Despite the significant advances made in the field of osteosarcoma treatment, the outcomes of this disease are still unfavorable. Besides, there is still no targeted therapy for osteosarcoma that can be used in clinical settings. Quercetin is a member of the phytochemical family which is used for different diseases including cardiovascular diseases, diabetes, and cancer. Its anti-cancer effects are examined in many types of cancer including breast, colon, lung, prostate, and pancreatic cancers and have shown promising results. Herein, the studies dealing with the antitumor roles of quercetin in osteosarcoma are reviewed in this article. We take a look into quercetin’s ability to affect proliferation, apoptosis, invasion, and chemo-resistance of the osteosarcoma cells through regulating protein expression and signaling pathways.

## **Background**

Osteosarcoma is a high-grade primary bone tumor that is defined by spindle cells originated from mesenchyme. Overall, osteosarcoma is a rare disease. However, it is the most common primary bone malignancy among children [1]. While this disease occurs sporadically, approximately 70% of tumor specimens show an abnormality in the chromosome. Moreover, regulation of cell cycle has been reported to demonstrate inherited defects in some cases [2]. In patients younger than 25 years old or older than 59 years, the age-adjusted incidence of osteosarcoma is 4 per 1 million people. However, this number drops to fewer than 2 per 1 million in people ages 25 to 59 years. The incidence of osteosarcoma is bimodal. The first peak occurs at the ages of puberty, implying the ages of 15 to 19 in boys and the ages of 10 to 14 in girls. The second peak occurs in the elderly with the age of 75 years [3]. Noteworthy, osteosarcoma is rare before the age of 5 [4]. With the application of multimodal chemotherapy, disease-free survival of patients with high-grade osteosarcoma has been improved to more than 60% compared to 10–20% which was reachable with the surgery as the only therapeutic approach. Currently, treatment of osteosarcoma is a combination of surgery and chemotherapy both before and after the surgery. Cisplatin, methotrexate, doxorubicin, and ifosfamide are common cytotoxic agents used for chemotherapy [5]. Although several chemotherapy regimens have been applied in the past 20 years, survival rates of patients are still not satisfying and no practical targeted therapy is discovered [6]. Therefore, it is important to investigate different therapeutic methods and anti-tumor agents in order to find an approach that provides a higher survival rate.

Quercetin is a common member of phytochemicals which can be found in daily foods, such as vegetables, nuts, and teas [7, 8]. Quercetin is a commercially accessible supplementary agent. It is reported that oral administration of 1 g quercetin per day is safe and is absorbed up to 60% [9]. Several studies have shown that quercetin plays a variety of pharmacological roles, including anti-proliferation, anti-oxidant, anti-inflammation, anti-microbial and anti-diabetes activities [8, 10–12]. Furthermore, quercetin is indicated to exert various anti-tumor effects both in vitro and in vivo against several cancers, such as ovarian cancer, colorectal cancer, lymphoma, gastric cancer, and breast cancer [13–17]. Herein, the studies dealing with the role of quercetin in the treatment of osteosarcoma are reviewed.

## Osteosarcoma pathogenesis

**Fig. 1:**
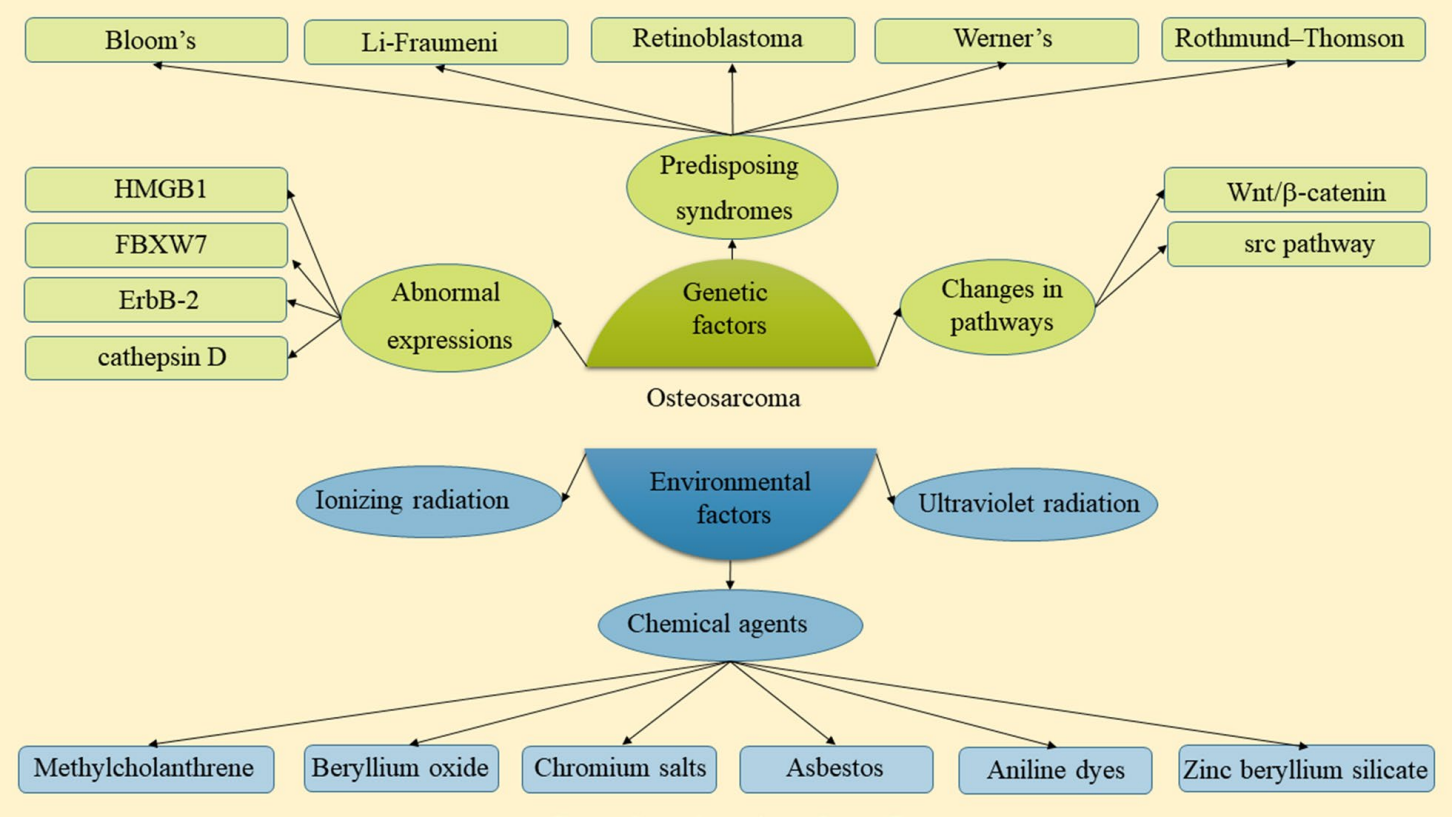
Factors involved in the pathogenesis of osteosarcoma can be divided into two types: environmental factors and endogenous factors. Endogenous factors include some predisposing syndromes (such as Li-Fraumeni syndrome), changes in cell signaling pathways, and abnormal expressions of proteins and RNAs. Meanwhile, environmental factors are mainly radiation and chemical agents

The molecular pathogeneses of osteosarcoma are heterogeneous (Fig. 1) [18]. Predisposition to osteosarcoma has been related to some syndromes, such as Li-Fraumeni syndrome, Retinoblastoma, Bloom’s syndrome, Werner’s syndrome, and Rothmund–Thomson syndrome [19–25]. The most common syndrome that predisposes to pediatric sarcomas is Li-Fraumeni syndrome in which the *TP53* gene is mutated in the germline. *TP53* encodes p53 which is a transcription factor regulating DNA repair genes and inducing post-damage apoptosis [26]. It is estimated that 30% of patients with Li-Fraumeni syndrome develop osteosarcoma. Furthermore, 18–26.5% of patients with sporadic osteosarcoma have shown somatic p53 loss [27, 28]. Retinoblastoma also leads to a predisposition to osteosarcoma. RB1 gene encodes retinoblastoma protein pRb which binds to the transcription factors of E2F family [29]. pRb loss occurs frequently in osteosarcoma sporadic cases and is associated with poor outcomes [30, 31]. Mutations in genes of RecQ helicases are also related to some rare autosomal recessive disorders, such as Rothmund–Thomson syndrome, Bloom’s syndrome, and Werner’s syndrome. These disorders are reported to be correlated to the higher osteosarcoma incidence [32].

In metastatic forms of osteosarcoma, some specific genetic changes have been observed which include upregulation of Wnt/β-catenin and src pathway, Notch1 and Notch2 receptors. Besides, downregulation of Fas/Fas ligand pathway (a cell death pathway) [33, 34]. Furthermore, angiogenic enzymes and growth factors (e.g. IL-8, PDGF-R, EGFR, and VEGF) are helpful for tumor progression and growth in target cells. Src pathway which is reactivated results in tumor hyper-proliferation and neovascularity [35, 36]. The heterogeneity in the genotype of osteosarcoma has translated into several expression profiles of macromolecular biomarkers which are helpful in the clinic. A variety of studies have found abnormally-expressed levels of certain proteins and mRNAs, such as HMGB1, ErbB-2, FBXW7, cathepsin D, and miR-421. Nevertheless, the significance of these biomarkers is still under investigation and controversy [37–41].

Among the environmental factors that are suggested as carcinogens of osteosarcoma, ultraviolet and ionizing radiation are well-established [42]. In only 2% of osteosarcoma cases, radiation exposure is implicated [43]. Moreover, data suggest that radiation does not play a significant role in the pediatric form of the disease. Between the exposure to radiation and the formation of osteosarcoma, there is an interval of 10–20 years [44]. Some chemical agents are also reported to be associated with the formation of osteosarcoma, including asbestos, methylcholanthrene, zinc beryllium silicate, chromium salts, aniline dyes, and beryllium oxide [45–48].

## Quercetin is a natural compound with a variety of advantages

The name of “Flavonoid” reminds us of a group of natural substances which are mostly found in vegetables [49]. These substances which encompass phenolic structures are classified into 6 subclasses: flavones, isoflavones, flavanones, flavonols, flavan-3-ols (flavanols), and anthocyanins [50]. Quercetin is a member of the flavonol group and is derived from *quercetum* (oak forest) [49, 51]. Apples, berries, onions, tea, tomatoes, and many seeds and nuts have quercetin as one of their ingredients [51, 52]. This polyphenol is one of the most investigated flavonols due to the diversity of its effects including anti-oxidative, anti-inflammatory, hepatoprotective, genoprotective, cytoprotective, and angioprotective [53, 54]. Investigations regarding the therapeutic applications of quercetin have shown its effectiveness on a number of diseases such as arthritis, allergy, diabetes, viral and bacterial infections, and finally cancer [53]. Before discussing the anti-cancer properties of quercetin, we would explain the mechanisms by which it is adsorbed, transferred, and metabolized inside the body.

The most common form of quercetin in nature is quercetin glycoside which is known to have poor bioavailability in the oral cavity [51]. In other parts of the gastrointestinal tract, quercetin adsorption is dependent on several factors especially the attached functional groups but the small intestine is the major adsorption site for quercetin glycosides [51]. Quercetin glycosides are deglycosylated in this site by the lactase phlorizin hydrolase (LPH) in order to form quercetin aglycone [55]. Afterward, quercetin aglycon enters stage II of the metabolism process [55, 56].

Quercetin gets metabolized in the small intestine through the xenobiotic metabolism which is made up of three stages: modification, conjugation, and elimination [57]. The metabolites of stage II, which are the results of glucuronidation, sulfation, and methylation, experience two different events: some of them are secreted into the portal and lymph circulation and some other metabolites go through elimination in the small intestine [53, 57]. In the liver, these metabolites get conjugated again and eventually, either enter the circulation or the bile [55]. Noteworthy, several factors involved in the regulation of these three stages of the quercetin metabolism are landmarks of its bioavailability [53]. The bioavailability of this agent is nearly 16% when ingested as a suspension and this poor bioavailability is mostly related to its absorption and biliary elimination [58]. However, a 44.8% bioavailability is also reachable for quercetin when administering quercetin aglycone solubilized in ethanol [59].

**Fig. 2:**
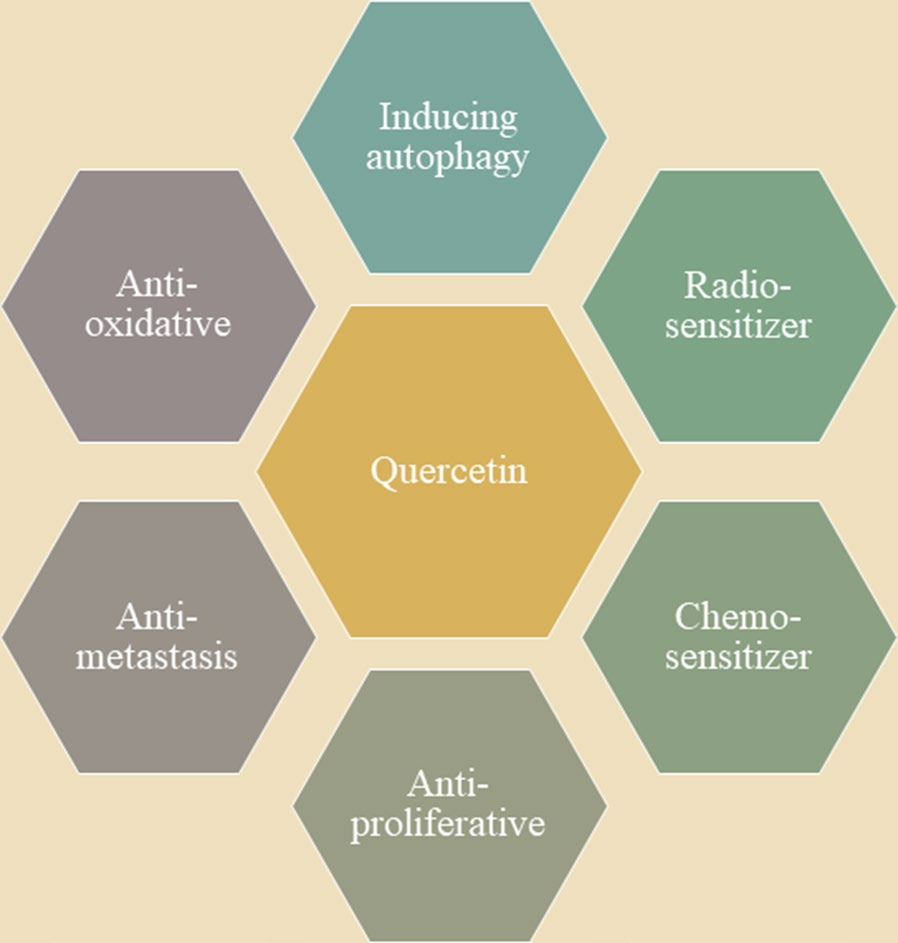
Quercetin beneficial roles in cancer treatment

After all, despite this feature of quercetin getting in the way of its applications, still, quercetin is considered an advantageous agent for therapeutic purposes. Investigations have approved its efficacy when used in a great number of diseases including cardiovascular diseases, diabetes, neurodegenerative diseases such as Alzheimer’s disease, arthritis, asthma, inflammatory bowel disease, and gastric ulcer [54, 60–69]. From a cancer point of view, quercetin affects many cancer hallmarks, such as proliferation, apoptosis, and autophagy, by the means of its properties (Fig. 2) [70–72]. For instance, quercetin is able to protect cells against oxidative stress by decreasing the number of reactive oxygen species (ROS) [70]. Subsequently, signaling pathways induced by ROS which are participating in cancer initiation/progression are inhibited by quercetin [70, 73]. In this regard, a great body of research has examined quercetin on different types of cancer. Anti-apoptotic effects of this favorable agent are observed in breast, colon, prostate, myeloma, pheochromocytoma, acute lymphoblastic leukemia, and ovarian cancer [74]. According to evidence, the vast majority of cancer types are prone to be affected by the anti-proliferative impacts of quercetin [75–77].

Additionally, quercetin also exerts some anti-metastasis effects through affecting inhibition of receptor for advanced glycation end products (RAGE) expression, c-MYC reduction, STAT3 signaling inhibition, inhibiting mesenchymal to epithelial transition (EMT), and increasing the invasiveness of the gastric, lung, bladder, and pancreatic cancerous cells [13, 78–81]. On the other hand, quercetin also has the capacity of targeting cancer cells in another way: chemo-sensitizing [82–85]. This effect of quercetin is mainly investigated in prostate cancer and it seems that it’s possible through regulating androgen receptor and PI3K/Akt signaling pathways [84]. Recent researches have demonstrated that radio-sensitizing is also detectable after quercetin treatment in bladder and colon cancer [86, 87]. After all, it seems that quercetin is a proper option for cancer treatment either alone or in combination with other therapeutic agents.

## Quercetin and osteosarcoma

**Fig. 3:**
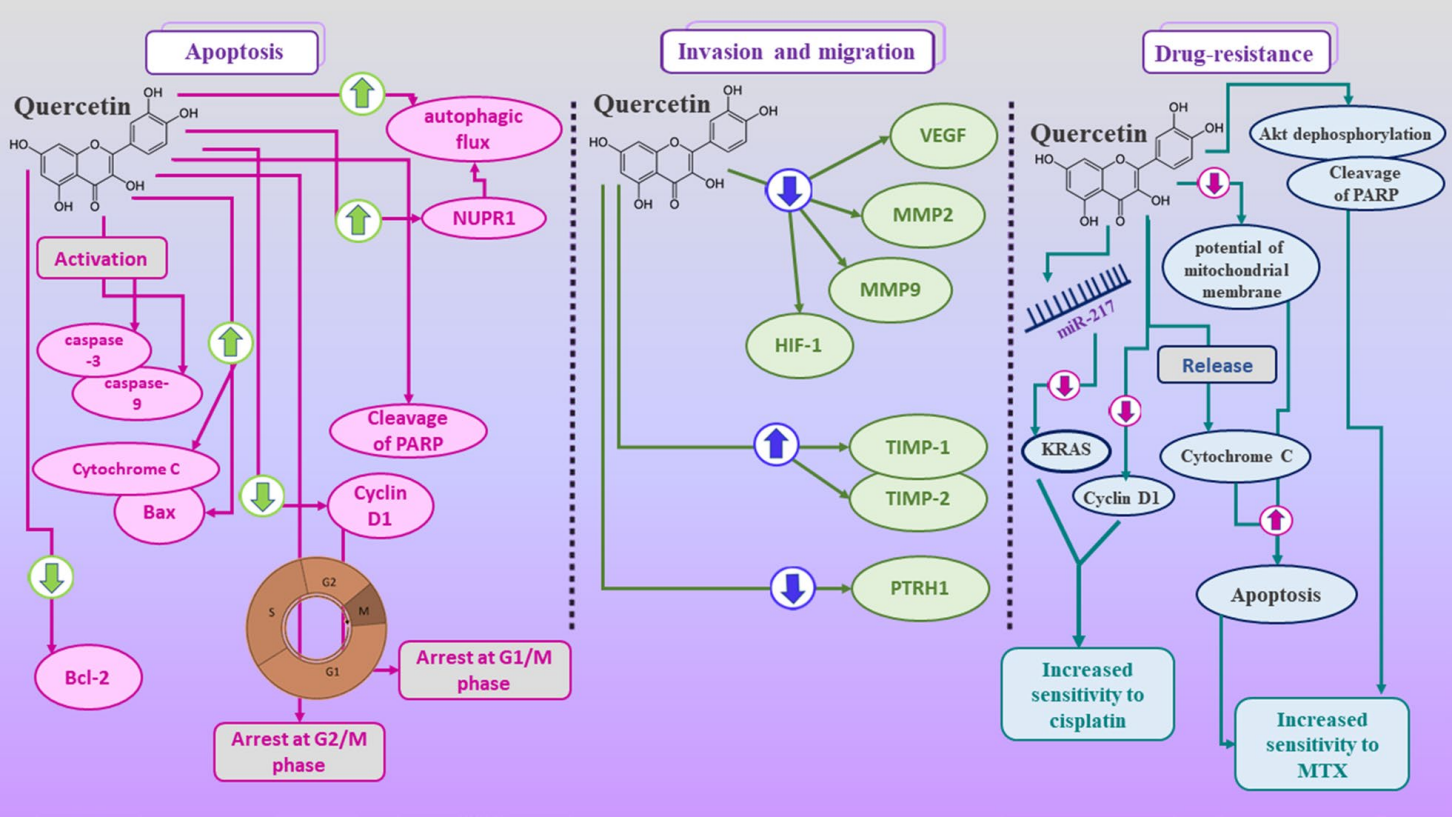
A summary of quercetin roles in osteosarcoma which include inducing apoptosis and suppressing proliferation as well as inhibiting migration and invasion. Furthermore, quercetin is shown to be effective in overcoming drug-resistance in osteosarcoma cells. To exert its anti-tumor roles, quercetin affects several molecular and cellular signaling pathways, such as VEGF, MMPs, caspases, AKT, and KRAS. Downward arrows represent downregulation or reduction. Upward arrows shows upregulation or increase

Studies have shown that quercetin plays a variety of antitumor roles against osteosarcoma (Table 1). Although these studies are mainly limited to in vivo and in vitro investigations, findings are promising (Fig. 3). In canine osteosarcoma cell lines, DSN and D-17, quercetin is indicated to reduce proliferation, change cell cycle and ROS levels, and increase apoptosis as well as altering the depolarization of mitochondria and calcium cytoplasmic concentration [88]. Besides, quercetin increases the phosphorylation of c-Jun N-terminal kinase, ERK1/2, P38, and P90RSK proteins. Meanwhile, it inhibits the phosphorylation of S6, AKT, and P70S6K proteins [88]. Evidence demonstrated that heat shock response leads to the reduction of glucocorticoid receptor binding activity in human osteosarcoma cell line HOS-8603 [89]. A study has shown that quercetin is able to suppress the mRNA expressions of heat shock protein (HSP)90α and HSP 70. However, it cannot abolish the reduction of glucocorticoid receptors during heat shock treatment. Also, it is found that quercetin-induced downregulation of glucocorticoid receptor is accompanied by a reduction in functional responses that are mediated by glucocorticoid [89].

**Table 1:** Studies investigated the antitumor roles of quercetin on human osteosarcoma

Dose(s)	Duration of incubation	Quercetin’s effect	Cell line(s)	Ref.
80 and 100 µM	48 h	Inhibits proliferation and metastasis through suppressing PTHR1	U2OS and Saos-2	[92]
25 or 50 µM	18 h	Suppresses proliferation and migration while inducing apoptosis	143B	[90]
25, 50 and 100 µM	12 and 24 h	Suppresses metastatic lung tumors	HOS and MG-63	[91]
10, 25 or 50 µM	48 h	Induces apoptosis by mitochondrial dysfunction and dephosphorylation of Akt	U2OS/MTX300	[94]
–	–	Reduces mitochondrial membrane potential and release of mitochondrial cytochrome c to cytosol while dephosphorylating Akt	U2OS/MTX300	[95]
50, 100 and 200 µM	24 h	Induces autophagy by the ROS-NUPR1 pathway	MG-63	[97]
10, 100, 200, 500 and 1000 µM	48 h	Induces apoptosis and cell cycle arrest at G(1)/S	HOS	[96]
20, 40, 80, 160, 240 and 320 µM	48 h	Induces apoptosis via a mitochondrial-dependent pathway and reduces cell viability	MG-63	[93]
–	–	Downregulated glucocorticoid receptors in osteosarcoma cells	HOS-8603	[89]
5 µM	24 h	Improves cisplatin sensitivity by miR-217/KRAS axis	143B	[98]
50 µM	48 h	Leads to the alteration in G1/S phase and reduction in cyclin D1 in U2OSPt	U2OS and U2OSPt	[99]

### Inhibiting proliferation, migration, and invasion

A study has also reported that quercetin treatment results in various antitumor effects in human osteosarcoma cell line 143B, including suppression of proliferation, cell cycle arrest at G2/M phase, induction of apoptosis, and reduced potential of cells for adhesion and migration [90]. Lan and colleagues have indicated that quercetin leads to a reduction in invasion and migration of osteosarcoma HOS and MG-63 cells [91]. They reported that quercetin-treated HOS cells show lower mRNA and protein levels of VEGF, HIF-1α, MMP2, and MMP9. Furthermore, the formation and growth of metastatic lung tumors are suppressed by quercetin treatment as studies in the nude mouse osteosarcoma model [91].

Quercetin treatment is shown to significantly reduce the cell viability of osteosarcoma U2OS and Saos-2 cells after 48 h of incubation [92]. Quercetin also significantly reduces the invasion, adhesion, and migration of cancer cells. It is reported that quercetin is able to decrease the expression of matrix metalloproteinases (MMP)-2 and -9 at the mRNA level. Meanwhile, it increases the mRNA expression of tissue inhibitors of metalloproteinases (TIMP)-1 and -2. 80 and 100 µM of quercetin leads to a significant reduction in parathyroid hormone receptor 1 (PTRH1) mRNA level by respectively, 0.27- and 0.55-fold in U2OS cells and 0.19 and 0.41-folds in Saos-2 cells. Furthermore, quercetin anti-tumor effects on osteosarcoma cell lines are improved by PTHR1 knockdown [92].

### Quercetin-induced induction of autophagy and apoptosis

Liang et al. [93] have found that quercetin inhibits the viability of human osteosarcoma MG-63 cells in a dose-dependent manner. They reported that quercetin treatment results in the activation of caspase-3 and -9, downregulation of Bcl-2, upregulation of Bax and cytochrome C, and loss of mitochondrial membrane potential. Based on this evidence, it is suggested that quercetin-induced apoptosis may be mediated by the mitochondrial-dependent pathway [93]. Another study has demonstrated that quercetin suppresses the viability of methotrexate (MTX)-resistant osteosarcoma cell line U2OS/MTX300 cells in a dose-dependent manner [94]. As evidenced by fluorescence staining and flow cytometry, quercetin induces apoptosis in the MTX-resistant cells, paralleled by a decrease in the potential of mitochondrial membrane, caspase-3 activation, mitochondrial cytochrome c release, Bax upregulation, and downregulation of p-Bad and Bcl-2. Followed by quercetin treatment, Akt dephosphorylation is observed. Active Akt plays a protective role against the Akt dephosphorylation, Bad, and degradation of poly(ADP-ribose) polymerase (PARP). Whereas, the combination of quercetin with LY294002 promotes the Bad and Akt dephosphorylation and cleavage of PARP [94]. The same result is concluded in a study by Yin et al. [95]. They reported that quercetin suppresses the proliferation and induces apoptosis in U2OS/MTX300 cells, suggesting that these might be associated with the apoptosis pathway of mitochondria and Akt activity [95]. Studies have also shown that quercetin treatment leads to the cell cycle arrest at G(1)/S phase accompanied by cyclin D1 downregulation [96]. Subsequently, caspase-3 activation and PARP cleavage induce apoptosis [96].

Quercetin’s ability to induce cell death in osteosarcoma cell lines is not limited to apoptosis. As shown by Wu and colleagues, incubation of MG-63 cells with quercetin for 24 h leads to an increase in autophagic flux [97]. Downregulation of P62/SQSTM1 and upregulation of LC3B-II/LC3B-I are confirming evidence of this effect. Using Bafilomycin A1, an inhibitor of autophagy, or blocking autophagy by knockdown of ATG5 causes a reduction in cell death induced by quercetin. Results indicate that quercetin treatment results in higher expression of NUPR1 and activation of NUPR1 reporter activity, leading to the expression of genes related to autophagy. Besides, NURP1 is reported to be associated with the dysregulation in the hemostasis of reactive oxygen species (ROS) which can be suppressed by NAC inhibiting intracellular ROS. Furthermore, in vivo studies reveal that quercetin-induced autophagy is suppressed by NAC [97].

### The role of quercetin in overcoming drug-resistance in osteosarcoma cell lines

5 µM quercetin is shown to increase the sensitivity of 143B cells to cisplatin treatment. Cisplatin and/or quercetin treatment leads to the upregulation of miR-217 and downregulation of KRAS, the target of miR-217, at both protein and mRNA levels. Knockdown of miR-217 abolishes the improved sensitivity to cisplatin. Meanwhile, overexpression of miR-217 leads to the opposite results, demonstrating that the miR-217-KRAS axis is involved in the quercetin-improved sensitivity of cisplatin [98]. Following the treatment with 50 µM quercetin for 48 h, the expression level of cyclin D1 is shown to be reduced in the cisplatin-resistance osteosarcoma cell line, U2OSPt; however, this did not occur in U2OS cells [99]. Moreover, it is reported that cyclin D1 decrease can be related to the changes in the G1/S phase following the quercetin treatment [99]. Altogether, these findings suggest that quercetin can be used alone as an anti-tumor agent or in combination with other cytotoxic agents as a synergistic compound.

## Conclusions

Osteosarcoma is a primary bone malignancy in both children and adults which is not considered to be common cancer. The number of newly diagnosed patients is not high but this fact has not affected its survival rate which recently has been enhanced to 60%. Other than the low efficacy of the common treatments, side effects of these methods are also interfering with the life quality of osteosarcoma patients especially the ones developing osteosarcoma at a young age. Considering this, increasing the effectiveness of common methods and decreasing their side effects would be a great help for osteosarcoma patients. Quercetin is a plant compound that has shown to be suitable for cancer treatment in recent years. Therefore, we tried to gather evidence on how quercetin is able to inhibit osteosarcoma in order to suggest a new candidate for the treatment of this cancer. In the osteosarcoma viewpoint, abundant effects of this agent have been indicated to be useful for inducing apoptosis, cell cycle arrest, and autophagy and reducing proliferation, viability, invasion, chemo-resistance, and migration (Figs. 1, 2 and 3). Nevertheless, for a complete confirmation of these effects and a wider usage in clinics, human studies in this field are required. As mentioned before, quercetin has a low bioavailability in the human body which increases the need for examining this agent on humans who are developing osteosarcoma. Moreover, the side effects of quercetin as an antitumor agent have not been fully investigated in previous studies and need to be addressed. Taken together, in this paper we have shown that quercetin executes a wide range of mechanisms for preventing osteosarcoma from progression; thus, it has the potential to become a common method in osteosarcoma management.

## Data Availability

Not applicable.

## Abbreviations

MMP: Matrix metalloproteinases
TIMP: Tissue inhibitors of metalloproteinases
MTX: Methotrexate
PARP: Poly(ADP-ribose) polymerase
ROS: Reactive oxygen species
HSP: Heat shock protein
LPH: Lactase phlorizin hydrolase
RAGE: Receptor for advanced glycation end products
EMT: Mesenchymal to epithelial transition
PTRH1: Parathyroid hormone receptor 1
